# Neurological Impact of SARS-CoV-2 Changing Variants: A 4-Year DW-MRI Study on Olfactory and Taste-Related Brain Regions

**DOI:** 10.3390/ijms26073164

**Published:** 2025-03-29

**Authors:** Teodora Anca Albu, Nicoleta Iacob, Daniela Susan-Resiga

**Affiliations:** 1 Department of Physics, West University of Timisoara, 300223 Timisoara, Romania; 2 ScanExpert, 300627 Timisoara, Romania; 3 Miclaus Diagnostic Hub SRL, 307200 Ghiroda, Romania; 4 Academia Romana Filiala Timisoara, 300223 Timisoara, Romania

**Keywords:** COVID-19 neurological involvement, olfactory and gustatory dysfunction, diffusion-weighted MRI, apparent diffusion coefficient, white matter microstructural changes

## Abstract

Neurological symptoms such as impaired smell and taste have been recognized as hallmark manifestations of severe acute respiratory syndrome coronavirus (SARS-CoV-2) infection. This study investigates and quantifies microstructural changes in the white matter of the olfactory bulb and taste-related brain regions (frontal operculum, insular cortex and parietal operculum) using diffusion-weighted magnetic resonance imaging (DW-MRI). Apparent diffusion coefficient (ADC) values were measured in patients with confirmed coronavirus disease of 2019 (COVID-19) at the onset of anosmia and ageusia (24 patients, scanned between March and December 2020), 1 month post-infection (20 subjects) and 36 months post-infection (20 participants). ADC values were analyzed over time and compared to normal white matter ADC ranges (calculated retrospectively from 979 pre-pandemic patients) and to those from patients infected with the 2024 strain of SARS-CoV-2 (27 patients). The results revealed significantly elevated ADC values in the white matter of the targeted brain regions, with a peak at the time of infection, followed by a decline 1 month post-infection, and a return to near-normal levels 3 years later. In contrast, the 2024 COVID-19 variant demonstrated reduced virus-related alterations in brain microstructure compared to the 2020 strain. These findings highlight the potential of DWI as a non-invasive tool for elucidating the molecular mechanisms underlying olfactory and taste dysfunction in COVID-19 patients.

## 1. Introduction

The COVID-19 pandemic, driven by the rapid spread of the SARS-CoV-2 infection, has had a profound global impact.

The earliest identified SARS-CoV-2 viral genomes were collected from patients in December 2019 in Wuhan, China. These genomes, including the reference genome *WIV04/2019*, were designated as the ancestral “A” type, with the derived “B” type following. The B type mutated into further types including B.1, which is the ancestor of the major global variants of concern, labeled with Greek letters in 2021 by the World Health Organization (WHO) as Alpha, Beta, Gamma, Delta and Omicron strains.

According to the European Centre for Disease Prevention and Control (ECDC), the Alpha variant (B.1.1.7), emerging worldwide in early 2020, was the first to be associated with the specific symptoms of COVID-19 that are discussed in this study. Viruses naturally acquire mutations over time, leading to new variants. For SARS-CoV-2, lineages often differ by only a few nucleotides. The Beta variant (B.1.351) was first demonstrated to be in circulation in May 2020 with very similar features in patients’ symptoms [1].

Ever since, SARS-CoV-2 has produced multiple strains and continues to spread globally, predominantly through descendant subvariants of the Omicron variant. Nowadays, the main circulating subvariant is XEC, which first appeared in Germany in June 2024 and has since spread rapidly throughout Europe [2].

In everyday clinical practice, distinguishing which COVID-19 variant a patient had acquired was challenging, as standard swab or polymerase chain reaction (PCR) tests indicated only positivity, not the specific strain. The only available way to infer variant prevalence was through WHO announcements regarding the emergence of new variants.

For this study, we assume that only the Alpha and Beta variants were circulating prior to October 2020 (when Delta was first reported), and that currently, only Omicron subvariants are prevalent.

Despite significant efforts to study the novel coronavirus, our understanding of many pathogenic aspects of COVID-19 remains limited. While respiratory symptoms are well known, neurological complications, such as anosmia (loss of smell) and ageusia (loss of taste), have also become increasingly recognized in COVID-19 patients with Alpha and Beta variants. Clinical and experimental studies have shown that various coronaviruses possess neurobiological abilities, spreading from the respiratory system to the central nervous system [3].

The hypothesis of SARS-CoV-2’s neurotropism is based on observations of sensory dysfunctions in COVID-19 patients, with evidence suggesting that these complications may indicate the virus’s neurovirulence. Numerous studies have explored the potential mechanisms behind COVID-19-induced smell and taste disorders [4,5,6]. One of the central theories is that the virus enters the olfactory system through the nasal epithelium, where it binds to angiotensin-converting enzyme 2 (ACE2) receptors, primarily found in non-neuronal cells [7]. This suggests that direct neuronal damage may not be the primary cause of dysfunction. In fact, recent studies propose that SARS-CoV-2 may trigger inflammation in the olfactory bulbs and tracts, leading to axonal damage and microvasculopathy, then spreading towards other parts of the brain, such as the gustatory cortex, which could explain anosmia and ageusia [8].

In assessing brain pathology, magnetic resonance imaging (MRI) has proven to be a valuable tool/pivotal. In terms of smell and taste disorders, several studies have reported olfactory bulb atrophy, dysfunctions or volumetric modifications in COVID-19 patients [9,10,11,12,13,14,15,16]. These imaging findings are considered indicative of viral neurotropism, the virus’s capacity to invade the nervous system, leading to structural brain alterations. However, the majority of studies examined the olfactory bulb exclusively, with less to no research conducted towards the gustatory cortex.

Among other MRI techniques, diffusion-weighted imaging (DWI) is particularly useful for studying highly cellular regions, as well as brain microstructure in various diseases, including COVID-19.

The DWI literature has reported brain diffusivity abnormalities in both gray and white matter in COVID-19 patients, but no research has targeted specific olfactory or gustatory regions [17]. Furthermore, DWI has been assessed by morphologic and visual evaluation only.

To quantify and objectively assess microstructural changes in various brain regions—too subtle to detect with the naked eye—apparent diffusion coefficient (ADC) maps are generated [18,19]. ADC values are calculated in a selected region of interest (ROI) from the maps. To address smell and taste discrimination using the ADC measurement approach, we condensed/diminished/reduced the searchlight analysis to the regionally selective olfactory and gustatory cortex.

**Figure 1:**
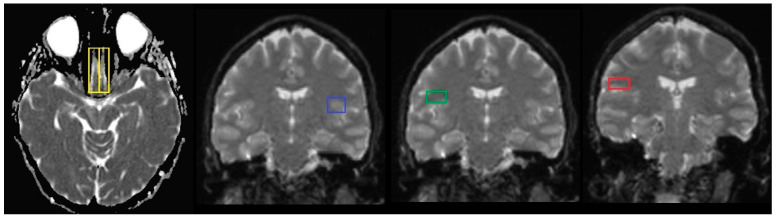
Theoretical placing of ROIs in the following areas: olfactory bulbs (yellow), insular cortex (blue), frontal operculum (green), parietal operculum (red).

The olfactory bulbs (OBs), responsible for the smell perception, are situated on the bottom side of the brain, one above each nasal cavity. The gustatory cortex (GC), responsible for the perception of taste, consists of the anterior insula (AI) in the insular lobe with its extending anatomically associated overlying frontal–parietal operculum (FO-PO) [20]. (Figure 1).

Olfactory and gustatory alterations have been noted as significant symptoms in COVID-19 patients, frequently appearing early in the disease course, often proving to be the sole features in otherwise asymptomatic patients, and then resolving in 4–6 weeks [21]. However, some patients experience persistent symptoms 12 months or even more post-infection, making it clear that long-term consequences occur (also known as long-COVID), hence the need for a follow-up at 24–36 months [22,23].

With the aim of clarifying the radiological findings of smell and taste alterations that are COVID-19-related in the short and long term, we performed extensive DWI research focusing on the specific olfactory and gustatory brain white matter area alterations in patients with clinically confirmed hyposmia/anosmia and hypogeusia/ageusia [24].

To our knowledge, no prior study has determined ADC values in olfactory or gustatory white matter regions, nor has any research tracked ADC brain microstructural changes over time in the same participants—during infection, 1 month post-infection, and 36 months later. Moreover, we reported on the first series of patients with COVID-19-induced olfactory and gustatory WM alterations, as confirmed by comparisons with previously available MRI.

Additionally, this study is the first to compare ADC measurements between the 2020 COVID-19 Alpha and Beta strain and the 2024 Omicron XEC variant. These findings could offer valuable insights into the mechanisms underlying COVID-19’s neurovirulence and potential differences in neurotropism over time.

## 2. Results

### 2.1. Pre-Pandemic Cohort

In total, 979 participants, who had radiological normal results, were retrospectively enrolled as control subjects, as they underwent MRI scanning before pandemic started in December 2019. In these normal brains, the mean ADC value in white matter, in general, was (0.752 ± 0.017) × 10^−3^ mm^2^/s, with a range of 0.710–0.797 × 10^−3^ mm^2^/s. The results are included in Table 1 under the label “pre-pandemic”. No significant differences were observed between the left (mean: 0.7523 ± 0.0143 × 10^−3^ mm^2^/s) and the right hemisphere (mean: 0.7525 ± 0.0147 × 10^−3^ mm^2^/s). Similarly, no statistically significant differences were identified between genders, with male participants showing a mean ADC value of 0.753 × 10^−3^ mm^2^/s and females 0.7515 × 10^−3^ mm^2^/s. Across age groups, mean ADC values remained stable at approximately 0.752 × 10^−3^ mm^2^/s until the age range of 40–50 years, at which point a slight increase was observed, with a mean of 0.760 × 10^−3^ mm^2^/s.

### 2.2. Acute Stage of 2020 COVID-19

In total, 24 COVID-19-positive patients exhibiting hyposmia or anosmia and hypogeusia or ageusia underwent DW-MRI scanning between May 2020 and October 2020 (only Alpha and Beta variants). Both sensory evaluations and MRI scans were conducted during the acute phase of infection, specifically between days 3 and 6. Results revealed that these patients had significantly higher ADC values compared to the control group in the white matter of the olfactory bulb (0.8489 ± 0.0113 × 10^−3^ mm^2^/s) and taste-related brain regions, including the frontal operculum (0.812 ± 0.0061 × 10^−3^ mm^2^/s), insular cortex (0.8259 ± 0.0082 × 10^−3^ mm^2^/s), and parietal operculum (0.7971 ± 0.0012 × 10^−3^ mm^2^/s). Meanwhile, in the distal white matter of the parietal lobe (chosen specifically for its anatomical distance from the olfactory and gustatory areas), ADC values were only slightly elevated, ranging between 0.755 and 0.789 × 10^−3^ mm^2^/s (mean value 0.772 × 10^−3^ mm^2^/s), in concordance with results from previous study [17] showing a mean value of 0.768 × 10^−3^ mm^2^/s.

### 2.3. One Month Post-Infection with 2020 COVID-19

One month after the initial examination, follow-up testing was conducted on 20 patients, including a second DW-MRI scan. This investigation revealed that all patients had recovered their sensory conditions. While olfactory and gustatory functions were fully regained and ADC values in the regions regulating these mechanisms drastically decreased, the values remained elevated compared to those of the control group, as follows: OB (0.7958 ± 0.0034 × 10^−3^ mm^2^/s), FO (0.7802 ± 0.0059 × 10^−3^ mm^2^/s), I (0.7836 ± 0.0038 × 10^−3^ mm^2^/s), and PO (0.7809 ± 0.0041 × 10^−3^ mm^2^/s).

### 2.4. Thirty-Six Months After Infection with 2020 COVID-19

Another DW-MRI follow-up was conducted with participants 36 months after the onset of COVID-19 symptoms. A total of 20 patients showed a decrease in ADC values compared to those in both the acute phase and at the one-month follow-up, with values approaching normal levels: OB (0.7595 ± 0.0064 × 10^−3^ mm^2^/s), FO (0.7482 ± 0.0020 × 10^−3^ mm^2^/s), I (0.7571 ± 0.0040 × 10^−3^ mm^2^/s), and PO (0.7519 ± 0.0025 × 10^−3^ mm^2^/s). The slight remaining difference may be attributed to natural aging, as ADC values in the control group also showed a small increase over time. Therefore, this finding falls within the normal range.

### 2.5. Acute Stage of 2024 COVID-19

In total, 27 COVID-19-positive patients underwent DW-MRI scanning between June and October 2024 during the acute phase (day 3–6 since symptom onset). Gustatory and olfactory tests revealed normal scores in most patients, with only slight alterations observed in two cases, resembling patterns seen in other viral respiratory infections (likely due to nasal congestion). While ADC values were elevated, they were significantly lower than those recorded in the acute cohort from 2020, as follows: OB (0.7893 ± 0.0008 × 10^−3^ mm^2^/s), FO (0.7858 ± 0.0018 × 10^−3^ mm^2^/s), I (0.7841 ± 0.0030 × 10^−3^ mm^2^/s), and PO (0.7817 ± 0.0043 × 10^−3^ mm^2^/s).

### 2.6. One Month Post-Infection with 2024 COVID-19

One month after the 2024 scans, a follow-up DW-MRI examination was performed on the same 27 participants. The results showed a rapid normalization of ADC values, decreasing to 0.7546 ± 0.0026 × 10^−3^ mm^2^/s in the olfactory bulb. In the gustatory cortex, values were 0.7611 ± 0.0026 × 10^−3^ mm^2^/s in the frontal operculum, 0.7665 ± 0.0034 × 10^−3^ mm^2^/s in the Insula and 0.7470 ± 0.0040 × 10^−3^ mm^2^/s in the parietal operculum. These findings suggest that the 2024 Omicron variant exhibits significantly less neurological involvement compared to the Alpha/Beta variants observed in 2020.

Mean (±standard deviation) ADC values are summarized in Table 1, with the aim to briefly outline reported values for targeted brain regions in different timeframes of COVID-19 illness.

These findings demonstrate a significant increase in ADC values within the olfactory and gustatory cortices during the acute phase of the 2020 COVID-19 variant’s spread, compared to pre-pandemic baseline values. The most affected regions included the olfactory bulb and the insula, followed by the frontal operculum and, to a lesser extent, the parietal operculum. This increase in ADC values aligns with the symptomatology reported by patients, including hyposmia or anosmia and hypogeusia or ageusia. Although a reduction in ADC values was observed 1 month post-infection, the levels had not yet returned to normal. Only at the 36-month follow-up did ADC values approximate those of the control group, suggesting a prolonged recovery process. The main outcome of the research arises from the connection (SPSS Pearson Correlation, correlation *p* = 0.0003) between the elevated ADC value and the infection phases (acute, and 1 and 36 months post-infection). Our results show a strong correlation: the more acute the infection, the more elevated the ADC values (SPSS Spearman’s rank correlation). These results highlight the neurobiological effects of the 2020 COVID-19 variant.

In contrast, the 2024 variant caused a slight elevation in ADC values in the acute phase, though these values were significantly lower than those observed with the 2020 variant. Additionally, the virus-related alterations of the 2024 variant appeared less aggressive, as the increase in ADC values was not accompanied by symptomatology. Furthermore, rapid recovery to normal ADC values was observed within one month post-infection.

## 3. Discussion

After four years of the pandemic and over 700 million cases worldwide, the neurological implications of SARS-CoV-2 infection remain a key area of research. Among the most common symptoms of acute COVID-19 are hyposmia or anosmia and hypogeusia or ageusia. However, the precise pathogenesis and molecular mechanisms underlying these olfactory and gustatory alterations remain unclear. The current literature suggests that SARS-CoV-2 can exert neurological effects, with certain variants demonstrating a greater potential to impact the nervous system than others. While a few studies have demonstrated radiological changes in the olfactory bulbs through dimensional and volumetric analyses or visual radiological interpretation, little attention has been paid to the gustatory cortex. MRI abnormalities associated with COVID-19 have largely been assessed using standard qualitative protocols, focusing on specific disease stages (e.g., the acute or hospitalized phase) or exclusively on the olfactory bulb.

The present study offers several key strengths: the use of quantitative diffusion-weighted imaging to analyze specific olfactory and gustatory brain regions, the integration of objective tools to evaluate olfaction and taste, longitudinal evaluations at periodic stages, and comparisons between pre-pandemic scans and those from the 2020 and 2024 COVID-19 variants.

The observed increase in apparent diffusion coefficient values in specific regions during the acute phase strongly supports the hypothesis that COVID-19 is neurotropic. A plausible explanation for these findings lies in the temporal dynamics of SARS-CoV-2 infection and its impact on the brain. During the acute phase, an immediate central nervous system response to viral aggression may influence water molecule diffusivity, resulting in elevated ADC values. While the most prominent changes are observed in the olfactory and gustatory cortices—aligning with the clinical manifestations of smell and taste dysfunction—subtle alterations may also occur in other brain regions. As demonstrated in our analysis of the distal parietal white matter, selected specifically for its anatomical distance from the olfactory and gustatory areas, ADC values were indeed slightly elevated during the acute phase. However, these values remained significantly lower than the mean ADC values observed in the olfactory and gustatory cortices. These findings further support our initial interpretation that the most pronounced microstructural changes are localized to regions directly involved in chemosensory processing. Nonetheless, the slight elevation observed in the remote white matter may suggest a broader effect of the systemic inflammatory response than initially assumed, warranting further investigation. The subsequent decrease in ADC values 1 month post-infection, and their near-normalization 36 months later, indicates the transient nature of these changes.

This study yielded four primary insights/findings:

1. Altered water molecule diffusivity in the white matter of the olfactory bulbs and gustatory cortex clearly correlates with the symptoms associated with the 2020 COVID-19 variant.
2. The most affected regions were the olfactory bulbs and the insula, followed by the frontal operculum, with the parietal operculum being the least affected.
3. There is no evidence of prolonged neurological effects in these targeted regions during chronic stages.
4. The 2024 variant demonstrated lower neurotropism, as evidenced by milder ADC changes and the absence of symptoms.

To our knowledge, this is the first study to publish quantitative DWI data on the olfactory bulbs and taste-related white matter regions (frontal operculum, insular cortex, and parietal operculum). A notable strength of this research is its longitudinal design, which investigates changes in diffusivity over time and provides definitive evidence of the transient nature of COVID-19-associated neurovirulence. This was achieved through multiple follow-ups with the same cohort, including pre-pandemic scans, acute-phase imaging of the 2020 COVID-19 variant, 1-month post-infection assessments, 36-month follow-ups, and imaging during and after the 2024 variant.

This study is the first to report data from such an extended longitudinal research design, encompassing comparisons across pre-pandemic, 2020, and 2024 conditions.

Future research could include comparative assessments of diffusion changes in various acute systemic infections—both with and without central nervous system involvement—as well as in other conditions affecting olfactory and gustatory function, such as head trauma or neoplasms. Such investigations would provide valuable insights into the specificity of the observed alterations and help differentiate localized effects from more generalized systemic responses. The existing literature indicates that DWI alterations are also observed in other viral infections, with acute disseminated encephalomyelitis (ADEM) serving as a notable example [25]. ADEM is characterized by an acute inflammatory response that produces multiple macroscopic lesions in the white matter, typically following viral infections. While specific ADC measurements in ADEM are not routinely reported across all studies, available data indicate diffusivity values in the range of 1.01–1.31 × 10^−3^ mm^2^/s (mean 1.24 ± 0.13 × 10^−3^ mm^2^/s) in the subacute stage (days 4–7). It is important to note that these ADC values are measured specifically within the macroscopic lesions, which naturally exhibit greater water diffusivity due to tissue disruption and edema. By contrast, the elevated ADC values observed in our COVID-19 cohort were recorded in normal-appearing white matter within the olfactory bulb (0.8489 ± 0.0113 × 10^−3^ mm^2^/s) and taste-related regions such as the frontal operculum (0.812 ± 0.0061 × 10^−3^ mm^2^/s), insular cortex (0.8259 ± 0.0082 × 10^−3^ mm^2^/s), and parietal operculum (0.7971 ± 0.0012 × 10^−3^ mm^2^/s), also assessed in the day 4–7 phase. Therefore, while the magnitude of ADC elevation in ADEM is expectedly higher due to overt lesion pathology, our findings highlight subtle yet regionally specific microstructural changes in COVID-19, even in the absence of overt lesions. In this way, the neurobiological alterations of SARS-CoV-2 refer to the neurological effects of the virus, including the associated changes in brain structures (such as the olfactory bulb and gustatory cortex), which can result from both direct viral effects and systemic inflammatory responses, rather than primary neuronal invasion. However, the alterations in ADC values are noteworthy because they suggest that even in the absence of clear neuronal damage, the inflammatory response and/or changes in blood–brain–barrier permeability can affect water diffusivity in these regions, which are involved in the sensory processing of smell and taste. Interestingly, even in healthy individuals, certain flavors are perceived through a combined contribution of taste and smell. For instance, the flavors of banana or coffee are largely experienced via olfactory input, as the brain integrates specific smells with associated taste perceptions. Further studies are warranted to explore how this interconnection between the gustatory and olfactory systems influences flavor perception and how it may be altered during infectious states, for example in cases of nasal congestion caused by rhinoviruses.

One limitation is the relatively small sample size, which reflects the challenges in following the same cohort over such a long period. Additionally, while this study provides valuable insights into the long-term impact of COVID-19 on olfactory and gustatory white matter regions, the 36-month follow-up could not entirely rule out possible reinfections, as this was assessed solely through patient-reported questionnaires.

## 4. Materials and Methods

### 4.1. Patient Recruitment

This study was approved by the Ethics Committee of Scientific Research of West University of Timisoara, Romania. The patients agreed and signed an informed consent form that followed the guidelines of the Declaration of Helsinki, to have their data stored and used for research purposes and to safely undergo the MRI examination. This study was approved by our institutional review Board.

Patients included in this study underwent MRI scans and were divided primarily into two groups. The before-COVID-19 group, comprising 979 subjects, was scanned before the onset of the SARS-CoV-2 pandemic. The COVID-19 group included the following subdivisions: 24 patients initially enrolled for serial imaging (during acute infection with the Alpha and/or Beta COVID-19 variants between May 2020 and October 2020), 20 of which were re-imaged at both 1 and 36 months post-infection; 27 participants scanned during acute infection with the Omicron XEC variant between July 2024 and October 2024; and the same 27 patients examined again one month after infection.

The general exclusion criteria for all participants, regardless of group (whether classified as normal or pathological), were as follows: inability to provide informed consent; claustrophobia; patients in a coma or under anesthesia; pediatric patients; lack of compliance; cognitive impairment; language barriers; the presence of motion or metallic or susceptibility artifacts; allergies to gadolinium-based contrast agents; a history of chemotherapy or radiotherapy; any neoplastic condition; or any neurologic or systemic disease potentially impacting the brain (e.g., diabetes, chronic obstructive pulmonary disease, hypertension, metabolic disorders, or dementia). Also, participants were excluded if they were taking any regular medication, except for topical treatments.

For the normal group, additional inclusion criteria stipulated that participants must have normal radiological findings, with no brain lesions (apart from minor age-related white matter changes, or leukoaraiosis, which were considered normal), and no family history of psychiatric disorders, dementia, or multiple sclerosis.

In the acute COVID-19 group, inclusion criteria required a positive COVID-19 diagnosis confirmed via a polymerase chain reaction (PCR) swab test or an over-the-counter rapid test on nasopharyngeal specimens. Participants also needed to exhibit hyposmia or anosmia and hypogeusia or ageusia, verified through the Sniffin’ Sticks battery test and the Gustatory Panel (assessing sweet and salty tastes), along with a classification of mild or moderate illness [26]. Enrollment in the acute group was limited to two specific periods: between May 2020 and October 2020, to include only Alpha and Beta variants, and between June and October 2024 for the current Omicron variant. For the follow-up COVID-19 group, rigorous verification ensured that participants were included within the designated timeframes of 1 month and 36 months post-infection.

### 4.2. Imaging Techniques

All imaging was conducted at Scan Expert Clinic, Romania, on a Siemens Magnetom Scanner (Siemens Medical Systems, Erlanger, Germany) at 1.5 Tesla using a standard head coil. The scanning protocol included axial and coronal diffusion-weighted (DW) images, alongside standard T2-weighted (sagittal and axial), 3D fluid-attenuated inversion recovery (FLAIR), and T1-weighted images (acquired axially and coronally, both before and after contrast administration). These images were used to rule out major brain pathology in normal brains and to identify potential alterations in COVID-19-related cases.

Diffusion-weighted imaging (DWI) derives its contrast by capturing variations in the movement of water molecules within tissues. Restricted water diffusion, typically due to interactions with cellular structures and macromolecules, correlates with increased tissue cellularity, as seen when abnormal cells limit the free movement of water molecules, causing a restricted appearance. Enhanced detection of these diffusion restrictions can be achieved by adjusting the amplitude, duration, and temporal spacing of diffusion-sensitizing gradients. These gradient settings define the “b-factor,” an essential parameter for DWI. For brain imaging, at least two b-values between 0 and 1000 s/mm^2^ are required; in this study, three values (0, 500, and 1000 s/mm^2^) were applied to generate DW images in three orthogonal directions. These images were then combined to create isotropic DW images and calculate apparent diffusion coefficient (ADC) maps on a pixel-by-pixel basis.

In this study, DW imaging was performed using a spin-echo echo-planar imaging sequence with a repetition time (TR) and echo time (TE) of 7000/103ms, capturing 40 slices of a 3 mm thickness with a 230 × 230 mm^2^ field of view, in both axial and coronal views. Diffusion measurements were taken in three orthogonal directions (x, y, and z) using b values of 0, 500, and 1000 s/mm^2^, with a total DW acquisition time of around four minutes each. This technique allows for rapid acquisition without requiring contrast agents, making it cost-effective and less sensitive to motion artifacts, and provides both qualitative and quantitative information.

Qualitatively, the visual representation of the lesion appears as a bright signal. The quantification aspect is less used in routine MRI evaluations and refers to the sensitivity to water motion arising in the DWI sequence through the apparent diffusion coefficient (ADC).

### 4.3. Data Analysis

**Figure 2:**
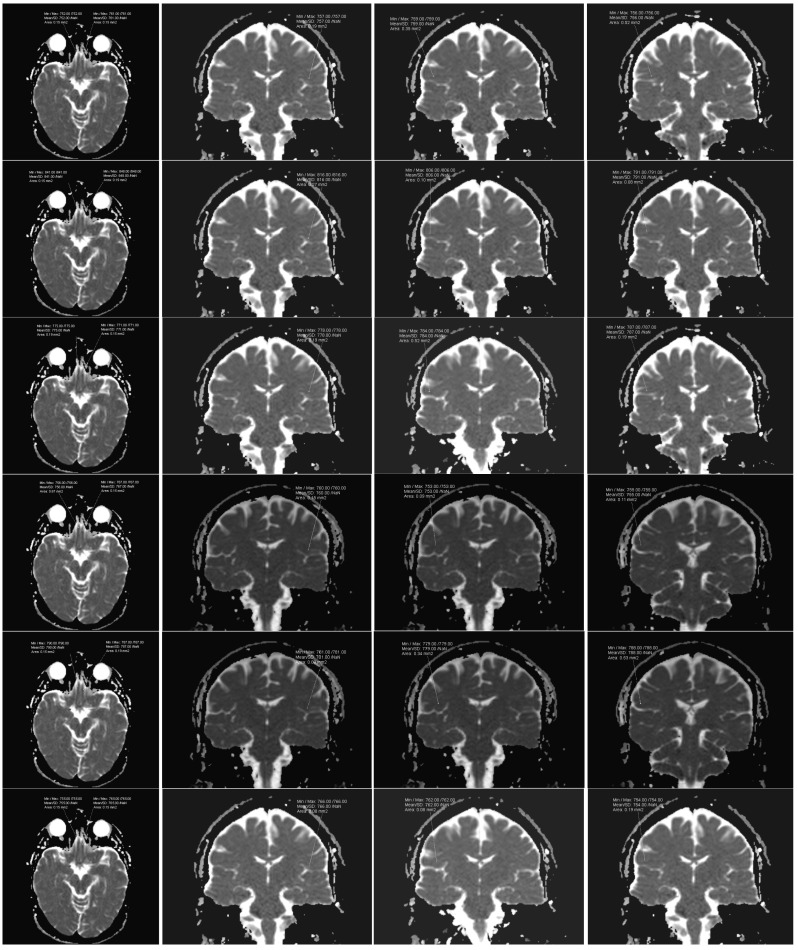
ROIs in the following areas: olfactory bulbs, insular cortex, frontal operculum, and parietal operculum for the normal cohort (1st row), those in the acute stage of 2020 variant (2nd row), individuals 1 month after infection (3rd row), individuals 36 months after infection (4th row), those in the acute stage of 2024 variant (5th row), and individuals 1 month post-infection (6th row).

DW images and their automatically generated ADC maps were transferred to a dedicated workstation for analysis. ADC values were obtained through post-processing by manually defining regions of interest (ROIs) on the ADC maps within the targeted tissue. Initially, ROIs were outlined on T2-weighted images (b = 0), where anatomical structures were more easily distinguishable. Next, these ROIs were then transferred onto the corresponding ADC images. For each ROI, measurements included the surface area and the minimum, maximum, mean, and standard deviation of the ADC values. ROI analysis was conducted using imaging analysis software (SyngoVia Plaza version VB10A, Siemens Medical Systems, Erlanger, Germany). The software enabled precise ROI placement as single-point measurements (circular in shape with minimal size), reducing the risk of including adjacent tissue, such as gray matter. In the normal group, ROIs were symmetrically drawn on white matter in both hemispheres. Concerning the COVID-19 category, ROIs were placed specifically within the white matter of the olfactory bulb and taste-related brain regions, including the insular cortex, frontal operculum and parietal operculum (Figure 2).

### 4.4. Statistical Analysis

The data obtained from ROI measurements were statistically analyzed using SPSS software version 16.0 (SPSS Inc., Chicago, IL, USA). The potential impact of COVID-19’s neurobiological alterations on ADC values was examined using Pearson and Spearman’s rank correlation. A one-way analysis of variance (ANOVA) was performed to compare mean ADC values within the normal group. Multivariate analysis of variance (MANOVA) was used to compare age cohorts across groups. Statistical significance was defined as a two-tailed *p*-value < 0.05.

## Figures and Tables

**Table 1:** Mean (±standard deviation) ADC values.

Pre-Pandemic	0.752 (±0.017)			
	OB	FO	I	PO
Acute Stage 2020	0.8489 (±0.0113)	0.812 (±0.0061)	0.8259 (±0.0082)	0.7971 (±0.0012)
1 Month	0.7958 (±0.0034)	0.7802 (±0.0059)	0.7836 (±0.0038)	0.7809 (±0.0041)
36 Months	0.7595 (±0.0064)	0.7482 (±0.0020)	0.7571 (±0.0040)	0.7519 (±0.0025)
Acute Stage 2024	0.7893 (±0.0008)	0.7858 (±0.0018)	0.7841 (±0.0030)	0.7817 (±0.0043)
1Month	0.7546 (±0.0026)	0.7611 (±0.0026)	0.7665 (±0.0034)	0.7470 (±0.0040)

## Data Availability

The data presented in this study are available on request from the corresponding author due to privacy reasons.

## Abbreviations

The following abbreviations are used in this manuscript:

SARS-CoV-2	Severe acute respiratory syndrome coronavirus 2
MRI	Magnetic resonance imaging
DWI	Diffusion-weighted imaging
ADC	Apparent diffusion coefficient
COVID-19	Coronavirus disease 2019
ECDC	European Centre for Disease Prevention and Control
PCR	Polymerase chain reaction
ACE-2	Angiotensin-converting enzyme 2
OB	Olfactory bulb
GC	Gustatory cortex
AI	Anterior insula
FO	Frontal operculum
PO	Parietal operculum
